# Proofreading neutralizes potential error hotspots in genetic code translation by transfer RNAs

**DOI:** 10.1261/rna.055632.115

**Published:** 2016-06

**Authors:** Jingji Zhang, Ka-Weng Ieong, Harriet Mellenius, Måns Ehrenberg

**Affiliations:** Department of Cell and Molecular Biology, Uppsala University, Uppsala 75124, Sweden

**Keywords:** ribosome, genetic code, accuracy, tRNA selection, proofreading, error hotspots

## Abstract

The ribosome uses initial and proofreading selection of aminoacyl-tRNAs for accurate protein synthesis. Anomalously high initial misreading in vitro of near-cognate codons by tRNA^His^ and tRNA^Glu^ suggested potential error hotspots in protein synthesis, but in vivo data suggested their partial neutralization. To clarify the role of proofreading in this error reduction, we varied the Mg^2+^ ion concentration to calibrate the total accuracy of our cell-free system to that in the living *Escherichia coli* cell. We found the total accuracy of tRNA selection in our system to vary by five orders of magnitude depending on tRNA identity, type of mismatch, and mismatched codon position. Proofreading and initial selection were positively correlated at high, but uncorrelated at low initial selection, suggesting hyperactivated proofreading as a means to neutralize potentially disastrous initial selection errors.

## INTRODUCTION

Recent biochemical observations on the accuracy of initial codon selection by seven aminoacyl-tRNAs (Johansson et al. 2012; Zhang et al. 2015) have raised pertinent questions. The data set displays a remarkable variation in the ability of aminoacyl-tRNAs in a ternary complex with EF-Tu and GTP to discriminate between codon–anticodon interactions with fully matching base pairs and those with a single mismatch. As expected, discrimination of uracil:guanine (U:G) mismatches in the middle codon position was comparatively poor. It was, at the same time, not expected that discrimination against the very same type of U:G mispairing in middle codon position would vary by two orders of magnitude depending on the type of tRNA in the ternary complex. Indeed, our recent findings are in stark contrast to previous claims that the accuracy of codon reading by tRNAs is nearly uniform (Gromadski et al. 2006). We also identified potential error hotspots for codon misreading in the living cell, like ${\text{Glu-tRNA}}_{\text{UUC}}^{\mathrm{Glu}}$ misreading codons GGA and GAU with predicted initial selection errors in the 1% range (Zhang et al. 2015). It was gratifying that the very same codons, GGA and GAU, had anomalously high errors also in vivo, as shown in pioneering work by Farabaugh and collaborators (Manickam et al. 2014). They used β-galactosidase mutants in which an essential glutamic acid (Glu) codon GAA had been replaced by near-cognate codons encoding other amino acids. Accordingly, the residual activity of the mutants depended on misreading of near-cognate codons by tRNA^Glu^ for errors above the background of the method, i.e., at error frequencies above the 10^−6^ to 10^−5^ range. It was at the same time clear that even in the low Mg^2+^ concentration limit our initial selection data (Zhang et al. 2015) fell short by factors of 10–100 in relation to the in vivo accuracy (Manickam et al. 2014), which includes the obligatory proofreading step (Thompson and Stone 1977; Ruusala et al. 1982; Gromadski and Rodnina 2004). A peculiar aspect that emerges through this comparison is that correspondence between in vivo and biochemical data requires proofreading to provide similar amplification factors as the total accuracy and initial selection change in the low accuracy range. Intuitively, however, one would expect initial selection and proofreading to co-vary, so high initial selection correlates with large factor of accuracy amplification by proofreading. The simplistic rationale is that the same type of codon–anticodon mismatch would be used for discrimination in both selection steps, which would lead to covariation of initial selection and proofreading.

In the present work, we explore the total accuracy of cognate in relation to all near-cognate codon reading by tRNA^Glu^, tRNA^Lys^, and tRNA^Phe^, in each case along with the proofreading contribution to the total accuracy level. By varying the Mg^2+^ concentration we were able to calibrate the accuracy level from our biochemistry to the codon reading accuracy in vivo monitored by Farabaugh and collaborators. We discuss the present remarkable result that proofreading decreases sharply with decreasing initial selection in the high initial selection accuracy range, but remains constant as initial selection decreases further in its low accuracy range.

The present result in combination with previous work has made our cell-free system for protein synthesis in vivo compatible with respect to all major steps of protein synthesis, including the accuracy of codon selection, initiation (Pavlov et al. 2011), peptide bond formation (Johansson et al. 2008a), translocation (Borg and Ehrenberg 2015), termination (Indrisiunaite et al. 2015), and ribosome recycling (Borg et al. 2015). This opens for extensive integration between in vitro and in vivo experiments, including systems biology modeling of bacterial physiology based on the biochemistry of protein synthesis. The close correlation that now exists between in vivo and in vitro kinetics of the ribosome may also serve as a guideline for biochemical experiments of relevance for living cells.

## RESULTS

### Measuring total accuracy of tRNA selection

**FIGURE 1.**
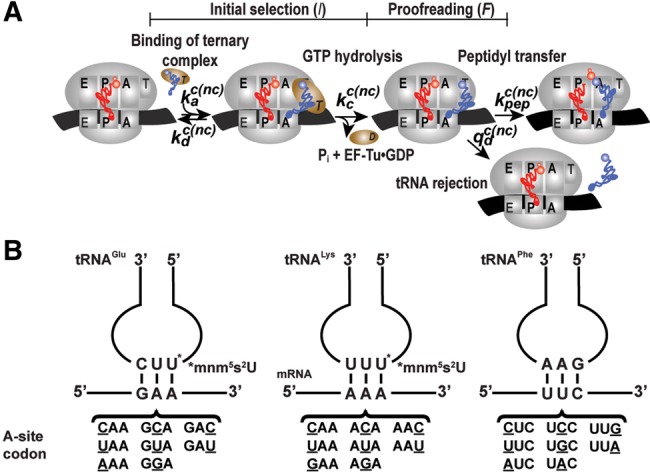
tRNA selection on the ribosome. (*A*) Kinetics scheme of tRNA selection on the mRNA-programmed ribosome. tRNAs are selected on the ribosome during initial selection (*I*) and proofreading (*F*) stages. These two stages were separated by GTP hydrolysis on EF-Tu. The total accuracy (*A*) of tRNA selection can be written as *A* = *I · F*. (*B*) The total accuracy of tRNA selection was measured for Glu-tRNA^Glu^, Lys-tRNA^Lys^, and Phe-tRNA^Phe^ reading all possible single-mismatch codons, compared to their fully matched codons AAA (tRNA^Lys^), GAA (tRNA^Glu^), and UUC (tRNA^Phe^). Mismatch codon positions are underlined.

Here we have used a cell-free system for protein synthesis with *Escherichia coli* components of high purity, specific activity, and in vivo-like kinetics (Johansson et al. 2008a) to study the accuracy by which three aminoacyl-tRNAs (aa-tRNAs) select their cognate in relation to all near-cognate codons on the messenger RNA (mRNA) programmed ribosome for subsequent peptide elongation. Ribosomes discriminate between cognate and noncognate aa-tRNAs in two consecutive steps, initial selection and proofreading. In this process, an aa-tRNA in a ternary complex with elongation factor Tu (EF-Tu) and guanosine triphosphate (GTP) enters a ribosome with a tRNA free A-site programmed with an amino acid encoding base triplet (codon) (Fig. 1A). During initial selection, the aa-tRNA in the ternary complex is selected in a reaction that proceeds to GTP hydrolysis with high probability if the codon is cognate and with low probability if the codon is near-cognate to the aa-tRNA. Proofreading selection occurs after dissolution of the ternary complex by GTP hydrolysis on EF-Tu, and the aa-tRNA proceeds to peptide bond with high probability if the codon is cognate but is ejected with high probability in the proofreading stage if the codon is near-cognate (Fig. 1A). It is generally assumed that proofreading occurs before aa-tRNA accommodation in the A site, as depicted in Figure 1A (Gromadski and Rodnina 2004; Ogle and Ramakrishnan 2005). It can, however, not be excluded that in some cases noncognate aa-tRNAs are discarded from the A site after accommodation but before peptide bond formation. The total accuracy, *A*, by which an amino acid is incorporated is defined by the ratio between the *k*_cat_/*K*_m_ values for peptide bond formation from a cognate (c) ternary complex and a noncognate (nc) ternary complex: *A* = (*k*_cat_/*K*_m_)^c^/(*k*_cat_/*K*_m_)^nc^. The universal definition of *k*_cat_/*K*_m_ for enzymatic reactions is as the rate constant for substrate association multiplied by the probability that the first enzyme–substrate encounter leads to product formation rather than substrate dissociation. In a steady state situation with equal concentrations of competing cognate and noncognate substrates the ratio between cognate and noncognate product formation flows is equal to the accuracy, *A*. For the experiments we used ${\text{Glu-tRNA}}_{\mathrm{UUC}}^{\mathrm{Glu}}$, ${\mathrm{Lys-tRNA}}_{\mathrm{UUU}}^{\mathrm{Lys}}$, and ${\text{Phe-tRNA}}_{\mathrm{GAA}}^{\mathrm{Phe}}$ with cognate codons GAA, AAA, and UUC, respectively, and near-cognate codons as shown (Fig. 1B). We prepared ternary complex mixtures with either one of the three aa-tRNAs in the ternary complex (T_3_) with EF-Tu and GTP. The mixtures in addition contained an energy regeneration system driven by phosphoenolpyruvate and other components, as described in Johansson et al. (2012). We prepared ribosome complex mixtures containing initiator tRNA charged with [^3^H]Met and formylated, ${f[}^{3}H]{\mathrm{Met}-\mathrm{tRNA}}_{\mathrm{CAU}}^{\mathrm{fMet}}$, in the initiation codon (AUG) programmed ribosomal P site, as described in Figure 1A (Johansson et al. 2012). The A site was programmed with any one of the cognate or near-cognate codons for the three aa-tRNAs (Fig. 1B). Incubation of the ribosome and ternary complex reactants for GTP hydrolysis in EF-Tu and subsequent peptide bond formation were started by mixing of the ribosome and ternary complex mixtures. The cognate reactions were in general fast and the reactions were carried out in a quench-flow instrument, as described in Johansson et al. (2012). The near-cognate reactions, however, were in general slow, so the experiments were carried out by hand.

**FIGURE 2.**
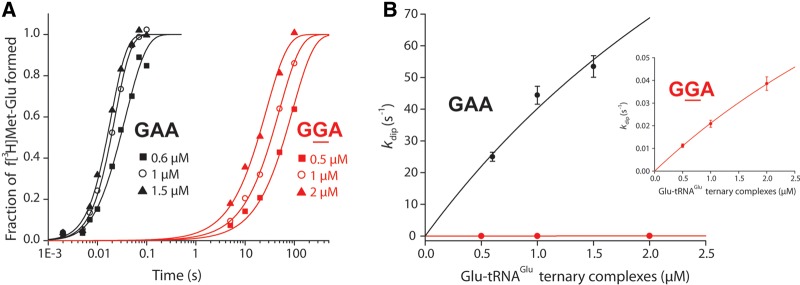
Measurements of cognate and near-cognate *k*_cat_/*K*_m_ values for dipeptide formation. (*A*) Time evolution of dipeptide f[^3^H]Met-Glu formation. Ternary complexes EF-Tu·GTP·Glu-tRNA^Glu^ were reacted with 70S initial complexes programmed with f[^3^H]Met-tRNA^fMet^ in the P site and a cognate codon GAA (black) or near cognate codon GGA (red) in the A site. Reactions were performed at increasing complex concentration as indicated in the figure. Ternary complexes were in excess over ribosomes so that the rate of dipeptide formation *k*_dip_ was limited by ternary complex concentration. (*B*) Concentration dependence of the rate of dipeptide formation *k*_dip_ estimated from *A*. *k*_cat_/*K*_m_ values were estimated by fitting the data into Michaelis–Menten model *k*_dip_ = ((*k*_cat_ / *K*_m_)[T_3_])/(1 + [T_3_]/*K*_m_). (*Inset*) Near-cognate reaction. Experiments were performed in polymix buffer with 2.3 mM free Mg^2+^.

In a typical set of experiments, a mixture with ${\text{Glu-tRNA}}_{\mathrm{UUC}}^{\mathrm{Glu}}$ in a ternary complex with EF-Tu ⋅ GTP was rapidly mixed in the quench-flow instrument (for the cognate reactions) or by hand (for the near-cognate reactions) with a ribosome mixture containing ribosomes A-site programmed with the cognate Glu codon GAA or the near-cognate Gly codon GGA. The reaction was quenched after different incubation times and the extent of f[^3^H]Met-Glu formation was monitored by HPLC with on-line radiometry, as described in Johansson et al. (2012). The extent of dipeptide formation at different T_3_ concentrations in excess is shown (Fig. 2A, black curves for the cognate and red curves for the near-cognate reactions). The cognate reactions took place in the 10-msec range and the near-cognate reactions in the 100-sec range. For both cognate and near-cognate reactions, the dipeptide formation rate increased with increasing ternary complex concentration (note the logarithmic time scale). The reaction rate, strictly defined as the inverse of the mean time for peptide bond formation, was plotted as a function of the ternary complex concentration (Fig. 2B, black curve in the cognate and red curve in the near-cognate case). The *k*_cat_/*K*_m_ and *K*_m_ values for both cognate and near-cognate reactions were estimated by fitting the Michaelis–Menten expression (Fig. 2B, legend) to the experimental curve (Fig. 2B; Supplemental Table S1).

### Calibration of cell-free codon selection accuracy to that in the living cell

**FIGURE 3.**
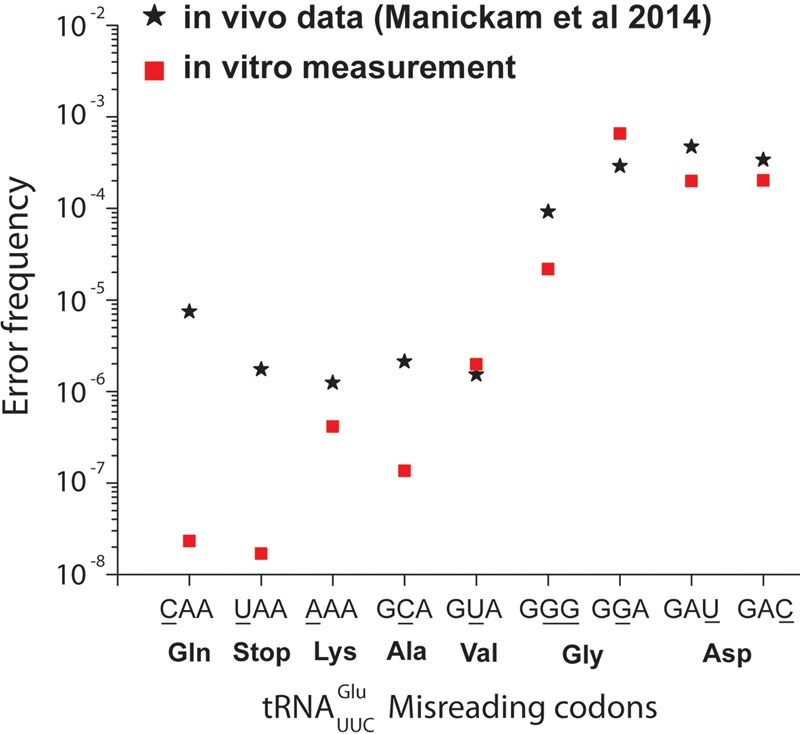
Comparison of in vivo and in vitro misreading error frequency. In vivo data (black stars) from the Farabaugh laboratory (Manickam et al. 2014) are based on induction of bioluminescence by mistranslation by tRNA^Glu^ ternary complex from *E. coli* strains with β-galactosidase mutants. In vitro measurements (red squares) were performed at 2.3 mM free Mg^2+^ and calibrated to in vivo condition according to the abundance of the two competing tRNA species in vivo (Dong et al. 1996) (see Materials and Methods) and assuming different ternary complexes as well as release factors have similar efficiencies for binding to ribosomes in the living cell. Mismatch codon positions are underlined.

Farabaugh and collaborators obtained estimates of the frequencies by which Glu-tRNA^Glu^ misreads codons CAA (Gln), UAA (stop), AAA (Lys), GCA (Ala), GGG (Gly), GGA (Gly), GAU (Asp), and GAC (Asp) in competition with the cognate tRNA readers of these codons in the living *E. coli* cell (Manickam et al. 2014). For this, they used a β-galactosidase (β-gal) mutant in which the functional Glu codon (GAA) at position 537 had been reprogrammed to each one of the above codons, near-cognate to Glu-tRNA^Glu^. Since Glu 537 is near-essential for β-gal function, β-gal activity could be used to estimate the tRNA^Glu^-dependent missense error frequency of Glu insertion at position 537. The caveat of an apparent misreading, caused by a background of residual β-gal activity of the mutated enzyme variants themselves or of wild-type enzymes emerging from errors in transcription or aminoacylation, was checked in experiments with hyper-accurate and error-prone ribosome mutants (Manickam et al. 2014). To calibrate the error level in our biochemical system to that in the living cell, we measured *k*_cat_/*K*_m_ values for peptide bond formation from Glu-tRNA^Glu^ containing a ternary complex on ribosomes programmed with all possible near-cognate codons. To derive the in vivo error frequencies, as measured by Farabaugh, from the present biochemical data, we used here the measured ratios of *k*_cat_/*K*_m_ values for cognate and near-cognate peptide bond formation, along with previous estimates of tRNA isoacceptor concentrations in the *E. coli* cell (Dong et al. 1996). This is motivated by previous findings that the variation of cognate *k*_cat_/*K*_m_ values is similar in the cases we have studied (Johansson et al. 2011; Zhang et al. 2015). The accuracy of our system was tuned by different concentrations of free Mg^2+^ ions from 1.3 mM (standard polymix concentration) to 7.5 mM (addition of 10 mM extra Mg^2+^ in polymix) (Johansson et al. 2012). Since there is no proofreading for the cognate codon reading (Thompson and Stone 1977; Ruusala et al. 1982; Gromadski and Rodnina 2004), *k*_cat_/*K*_m_ values are the same for GTP hydrolysis and peptide bond formation. At each Mg^2+^ concentration, we used the *k*_cat_/*K*_m_ values for GTP hydrolysis from the ternary complex with Glu-tRNA^Glu^ on ribosomes programmed with Glu codon GAA under the same condition from Zhang et al. (2015) as a proxy for in vivo reading of all codons near-cognate to the Glu codon by their respective cognate tRNAs and release factors (see Materials and Methods). The cognate and near-cognate *k*_cat_/*K*_m_ values for fMet-Glu formation were measured at each Mg^2+^ concentration as described in the previous section (Supplemental Table S1). The best accuracy fit with in vivo accuracy was obtained at 2.3 mM free Mg^2+^ concentration, as shown in Figure 3. Comparison between our data from biochemical experiments (red squares) and in vivo data (black stars) shows good correlation at error levels from 10^−5^ and higher. It is also clear that the error hotspots for Glu-tRNA^Glu^ reading of the near-cognate Gly (GGA) and Asp (GAU, GAC) have been independently identified in vivo (Manickam et al. 2014) and in the present work, highlighting the physiological relevance of our biochemistry. It is seen that when our error estimates go down below 10^−6^, the in vivo estimates remain in the 10^−6^ to 10^−5^ range, a result very likely due to an error background in this range (Manickam et al. 2014).

### Accuracy of genetic code translation varies over five orders of magnitude

**FIGURE 4.**
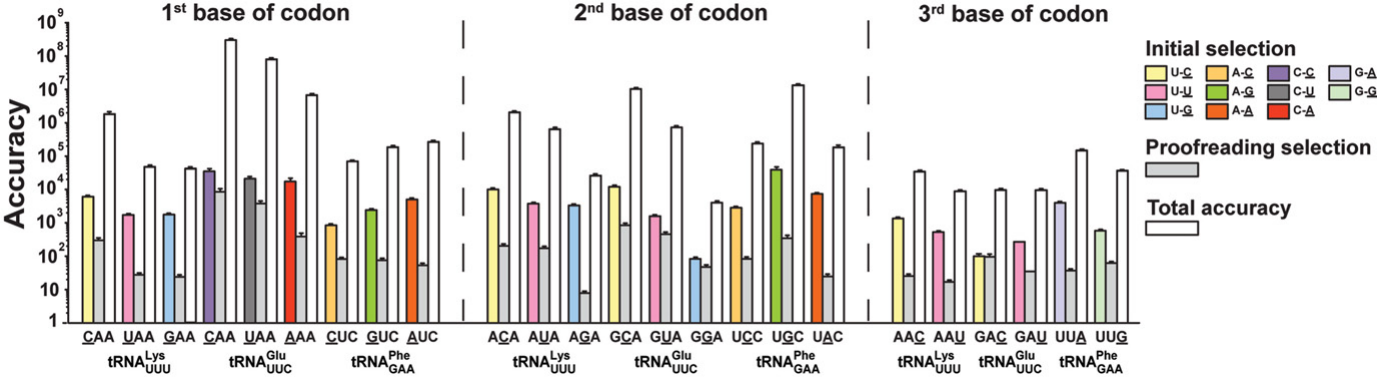
Accuracy of tRNA selection varies with mismatch codon positions, type of mismatch, and type of aa-tRNA. Total accuracy for single-base mismatch codons reading by tRNA^Lys^_UUU_, tRNA^Glu^_UUC_, and tRNA^Phe^_GAA_ were summarized with respect to mismatch codon positions and mismatch identities. First, second, and third codon mismatch positions are shown in *left*, *middle*, and *right* panels, respectively. Mismatch identities are shown in different colors for initial selection. Gray area for each mismatch shows the contribution of proofreading (*F*) to total accuracy (with the white area), where proofreading was calculated from overall accuracy divided by initial selection (see Materials and Methods). Measurements were performed at 2.3 mM free Mg^2+^. Initial selection data were from Zhang et al. (2015).

Next, we expanded the total accuracy measurements to include also fMet-Lys and fMet-Phe formation from Lys-tRNA^Lys^ and Phe-tRNA^Phe^ containing a ternary complex respectively on ribosomes with cognate and all near-cognate codons for these two aa-tRNAs (Fig. 1B). All measurements were performed at 2.3 mM free Mg^2+^ concentration (addition of 2 mM extra Mg^2+^ in polymix) and, hence, under conditions relevant to the corresponding accuracy levels in the living cell (Fig. 3). As described above, the accuracy was in each case obtained as the ratio between the *k*_cat_/*K*_m_ value for a particular tRNA isoacceptor reading its cognate codon and the *k*_cat_/*K*_m_ value for the same tRNA reading a near-cognate codon. All *k*_cat_/*K*_m_ values for dipeptide formation on Glu-tRNA^Glu^, Lys-tRNA^Lys^, and Phe-tRNA^Phe^ are summarized in Supplemental Table S1. The accuracy values are shown graphically in Figure 4 (white staples) and summarized in Table 1.

**TABLE 1.**
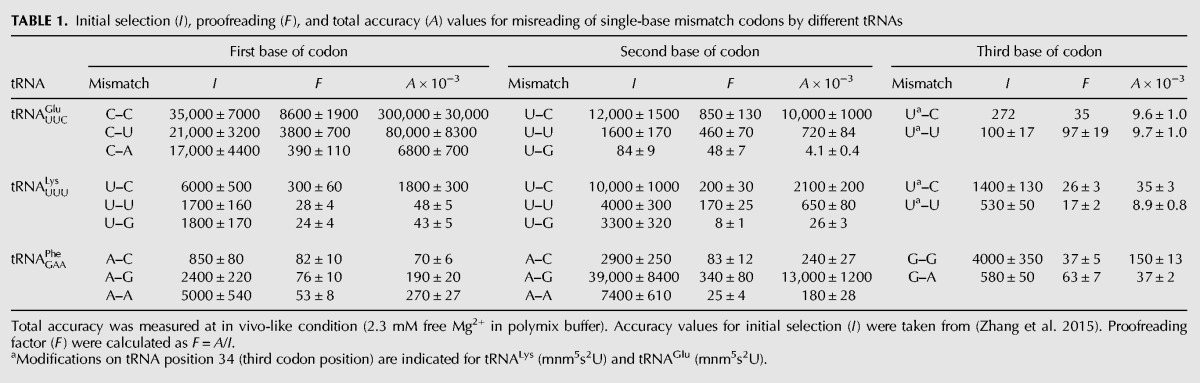
Initial selection (*I*), proofreading (*F*), and total accuracy (*A*) values for misreading of single-base mismatch codons by different tRNAs

### How proofreading correlates with initial selection

In bacterial translation, the total accuracy, *A*, is factorized into an initial selection part, *I*, and a proofreading part, *F* (Fig. 1A; Thompson and Stone 1977; Ruusala et al. 1982): *A* = *I* × *F*. Previously, initial selection values (*I*) were estimated from the ratio of *k*_cat_/*K*_m_ values for GTP hydrolysis between cognate and near-cognate reactions (Johansson et al. 2012; Zhang et al. 2015)*.* Using initial selection values obtained at a concentration of free Mg^2+^ of 2.3 mM (Zhang et al. 2015), we calculated the proofreading factor, *F*, from the initial selection, *I*, for every accuracy, *A*, through *F* = *A*/*I* as shown graphically in Figure 4 and summarized in Table 1. There is a remarkably large variation in the accuracy of codon reading depending on tRNA identity, mismatch position in the codon, and type of mismatch. The accuracy varied by five orders of magnitude, from 4 × 10^3^ to 3 × 10^8^, initial selection and proofreading by about three orders of magnitude from 100 to 40,000 and eight to 8000, respectively (Table 1; Fig. 4).

**FIGURE 5.**
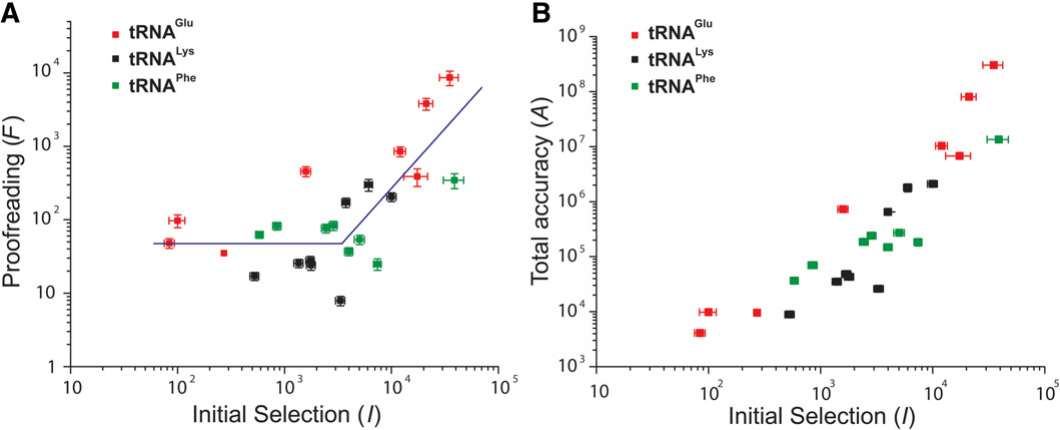
Enhanced proofreading on initial selection error hotspots. (*A*) Correlation between proofreading (*F*) and initial selection (*I*): a linear fit of log_10_(*F*) versus log_10_(*I*) for high *I*-values with a slope of 1.6 for *I*-values above 10^3.5^; at low *I*-values, in contrast, log_10_(*F*) remains virtually constant at 1.7 as log_10_(*I*) decreases further. A full description of the statistical analysis is found in the Supplemental Material. (*B*) Correlation between total accuracy (*A*) and initial selection (*I*). Data were obtained for all possible single-base mismatch codons reading by tRNA^Glu^_UUC_, tRNA^Lys^_UUU_, and tRNA^Phe^_GAA_. Measurements were performed at 2.3 mM free Mg^2+^. Initial selection data were from Zhang et al. (2015).

It is often assumed that initial selection and proofreading have straightforward dependence on the very same type of codon–anticodon interaction to discriminate between cognate and noncognate codon reading. This would mean similar dependence of *F* and *I* on the same intrinsic discrimination parameter *d* and suggest that log_10_(*F*) formally plotted as a function of log_10_(*I*) would be a straight line with slope one. The parameter *d* is defined as the highest possible substrate selection accuracy of an enzyme state, as obtained when the rate constant of the forward reaction is much smaller than that of the discard reaction for both cognate and noncognate substrates (Ehrenberg and Blomberg 1980; Johansson et al. 2012; Zhang et al. 2015). What is observed, however, is a straight line with slope 1.6 at log_10_(*I*) values larger than 3.5 and virtually constant log_10_(*F*) at 1.7 in response to decreasing log_10_(*I*) values smaller than 3.5 (Fig. 5A) (see Discussion and Supplemental Material).

The division of initial selection into a high and a low selection range was motivated by a comparison of the curve fitting with one line for the whole accuracy range (adjusted *R*^2^ value of 0.31; Supplemental Fig. S1; Supplemental Table S2), one constant and a line (adjusted *R*^2^ value of 0.55; Fig. 5A; Supplemental Fig. S1; Supplemental Table S2), or two lines (adjusted *R*^2^ value of 0.53; Supplemental Fig. S1; Supplemental Table S2). The hypotheses that (1) no linear correlation at all is better than the straight line correlation, (2) the straight line is a better model than the constant intersecting with a straight line, and (3) the constant and straight line model is better than a model with two intersecting straight lines, were all tested using the *F* distribution. The statistical analysis showed that hypotheses (1) and (2) could be rejected with *P*-values of 0.0108 and 0.00187, respectively, but that hypothesis (3) could not be rejected. These results and the adjusted *R*^2^-values (Supplemental Table S2) suggest that the best model is a constant and a straight line. For the full statistical analysis, see Supplemental Material.

## DISCUSSION

**FIGURE 6.**
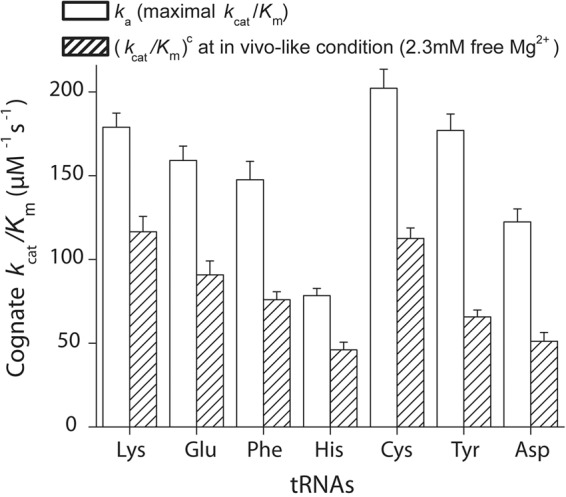
Kinetic loss for cognate reaction under in vivo condition. Cognate kinetic efficiency (*k*_cat_/*K*_m_)^c^ under in vivo condition (at 2.3 mM free Mg^2+^, striped areas) for different tRNAs was compared to the association rate constants for binding of ternary complexes to the ribosome *k*_*a*_, which is the maximal kinetic efficiency for dipeptide formation. All data shown here are from Zhang et al. (2015).

In this work, we have collected a data set comprising the total accuracy and its partitioning in initial selection and proofreading by which each one of three of the 54 tRNA isoacceptors in *E. coli* discriminates between its cognate codon, containing three Watson–Crick base pairs, and all near-cognate codons containing one mismatched base pair (Table 1; Fig. 4). The accuracy of the data set was calibrated to the accuracy of codon reading in the living cell by varying the free Mg^2+^ concentration in the biochemical assays for best correspondence with the accuracy by which Glu-tRNA^Glu^ discriminates against codons near-cognate to the Glu codon (GAA). As mentioned above, the in vivo data set was obtained by careful measurement of the residual activity of β-galactosidase mutants expressed from open reading frames in which a Glu codon had been altered to codons near-cognate to Glu-tRNA^Glu^ (position 537) (Manickam et al. 2014). The best correspondence between in vivo and in vitro data was obtained for a free Mg^2+^ concentration of 2.3 mM in the polymix buffer previously developed for fast speed and high accuracy of mRNA translation (Johansson et al. 2012). At this Mg^2+^ concentration, the *k*_cat_/*K*_m_ values for tRNA^Glu^, tRNA^Lys^, and tRNA^Phe^ were reduced about twofold in relation to their maximal values, as determined by their respective association rate constants (Fig. 6; Zhang et al. 2015). This may seem like a high kinetic cost (Gromadski and Rodnina 2004; Gromadski et al. 2006), but it must be borne in mind that increasing the Mg^2+^ concentration to speed up the cognate kinetics will activate near-cognate and noncognate ternary complexes as inhibitors of cognate protein synthesis (Johansson et al. 2008b). This means, in other words, that what appears as a gain in reality is a kinetic loss. We further note that the rate of translocation decreases sharply with increasing Mg^2+^ concentration (Borg and Ehrenberg 2015) and that, finally, 2.3 mM is close to estimates of the free, intracellular Mg^2+^ concentration (Alatossava et al. 1985).

The calibration attempt endeavored here is a multiparameter problem. It is therefore unlikely that the present approach will provide perfect in vivo mimicking conditions. One reason is that there is a multitude of buffer components in the living cell that are absent in the polymix buffer, and for many of the common components the free concentrations have remained unknown. Another reason is that kinetics in the living cell proceeds under conditions of very high protein density (McGuffee and Elcock 2010), affecting the speed of all diffusion-controlled reactions, whereas kinetics in the test tube proceeds in dilute solution.

It should, at the same time, be stressed that calibration of speed and accuracy of cell-free and intracellular protein synthesis and protein synthesis in the living cell have not been previously achieved. We deem such a calibration essential for two main reasons. The first is that when in vitro and in vivo kinetics match each other, then biochemical modeling can root bacterial physiology and population genetics in kinetically well-characterized steps of ribosomal protein synthesis and its auxiliary reactions. Such rooting will, not the least, facilitate deep understanding of resistance development against antibiotic drugs. The second reason is that when the biochemistry of bacterial protein synthesis is calibrated to its intracellular counterpart, the relevance of the functional observations in the test tube will be much easier to assess. When the relevance is ascertained it will become even more meaningful to discuss the structures of the ribosome and its auxiliary factors in terms of the evolutionary pressure that has shaped their kinetics within the constraints of physical law.

The accuracy of near-cognate codon reading displays an enormous variation (Table 1; Fig. 4), which we suggest to be at work also in the living cell, as seen here in a special case (Fig. 3). Indeed, from our data we propose that the average intracellular error frequency is dominated by a small number of error hotspots similar to those for tRNA^Glu^ misreading near-cognate codon U:G in second and U:U in third codon position. Another is likely to be tRNA^His^ misreading U:G in second or third codon position (Zhang et al. 2015). This unevenness also motivates qualification of the common wisdom that the error frequency of transcription is much lower than that of translation (Gout et al. 2013; Imashimizu et al. 2013). It now seems that there is strong, template context-dependent transcription error variation (Mellenius and Ehrenberg 2015), and this prediction in conjunction with the present data (Table 1) suggest that transcription errors dominate greatly in some and translation errors in other contexts.

A striking feature of our data set is that initial selection and proofreading are strongly correlated in the high but not in the low initial selection range (Fig. 5A). That is, a plot of the logarithm of the proofreading parameter, *F*, versus the logarithm of the initial selection, *I*, is a straight line with a steep slope for high *I*-values. At low *I*-values, in contrast, log_10_(*F*) remains virtually constant as log_10_(*I*) decreases further, so the total accuracy decreases much more slowly with *I* in this region (Fig. 5B). One consequence of this is that what initially appeared as a catastrophic decline in initial selection by tRNA^Glu^ reading G in second and U or C in third position (Zhang et al. 2015), can now be seen to have misreading frequencies below the “canonical” in vivo error frequency of 1/2000 (Parker 1989, 1992; Kurland 1992). To explain this we first note that the large local variation of log_10_(*F*) as a function of log_10_(*I*) is not caused by measurement errors, which are confined to a 15% range, but to a real variation as it exists in living cells. At high initial selection values, the total accuracy is so high that decreasing *d*-values in initial selection and, by hypothesis, proofreading (Ehrenberg and Blomberg 1980; Johansson et al. 2012; Zhang et al. 2015) do not confer significant fitness reduction and are therefore tolerated. When, however, the *d*-values of initial selection and proofreading are reduced below an accuracy threshold, at which protein synthesis errors would greatly reduce the quality of the proteome and significantly decrease the fitness of the bacterial population, the evolutionary response has been to increase the expression of the *d*-values of proofreading. In a simple model this is done by reducing the ratio between the forward and discard rate constants in the proofreading branches (Supplemental Material). If taken at face value, the observation that the slope of the log_10_(*F*) variation with log_10_(*I*) is 1.6 (Fig. 5A) may suggest proofreading in two steps (Ehrenberg and Blomberg 1980) rather than one as previously suggested (Thompson and Stone 1977; Ruusala et al. 1982; Gromadski and Rodnina 2004; Gromadski et al. 2006). Indeed, two-step proofreading is a particularly attractive hypothesis since it would provide a natural explanation of how the expression of proofreading for noncognate substrates can be cranked up at low *d*-values with small concomitant losses of kinetic efficiency for the cognate reactions (Ehrenberg and Blomberg 1980). This being said, it is also clear that much more direct experimental evidence would be necessary to prove the existence of multistep proofreading of transfer RNAs in genetic code translation among bacteria.

## MATERIALS AND METHODS

### Regents and buffer conditions

The purified *E. coli* translation system, including 70S ribosomes purified from MRE600 strain, synthetic mRNAs, initiation factors, elongation factors, and f[^3^H]Met-tRNA^fMet^, were prepared according to Johansson et al. (2008a) and references therein. *E. coli* tRNA^Glu^, tRNA^Lys^, and tRNA^Phe^ were from Chemical Block. [^3^H]Met were from PerkinElmer, and all other chemicals were from Merck or Sigma-Aldrich. All experiments were performed in polymix buffer (Jelenc and Kurland 1979) containing 95 mM KCl, 5 mM NH_4_Cl, 0.5 mM CaCl_2_, 8 mM putrescine, 1 mM spermidine, 5 mM potassium phosphate, 1 mM dithioerythritol, 10 mM phosphoenolpyruvate (PEP), 5 mM Mg(OAc)_2_, 1 mM ATP, and 1 mM GTP, with Mg(OAc)_2_ additions between 0 and 10 mM, and supplemented with 1 µg/mL pyruvate kinase and 0.1 µg/mL myokinase for energy regeneration. As PEP chelates Mg^2+^ with a *K*_d_-value of 6 mM (Wold and Ballou 1957) and assuming that one ATP or GTP molecule chelates one Mg^2+^, the free Mg^2+^ concentration in the reaction was estimated to be between 1.3 mM [without addition of Mg(OAc)_2_] to 7.5 mM [with 10 mM addition of Mg(OAc)_2_]. Free Mg^2+^ concentration was estimated to be 2.3 mM when 2 mM of extra Mg(OAc)_2_ was added to polymix buffer.

### Measurement of cognate and near-cognate dipeptide formation

Final concentrations after mixing equal volume of ribosome and ternary complex mixtures are given below. All kinetics measurements were performed at 37**°**C. Ribosome mixture, containing ribosomes programmed with mRNA with mismatch A-site codon, were prepared by incubating 70S ribosomes (at concentrations at least 2× lower than ternary complex concentrations and between 0.2 and 0.5 µM), f[^3^H]Met-tRNA^fMet^ (1.5× ribosome concentration), mRNA (2× ribosome concentration), IF1 (1.5× ribosome concentration), IF2 (0.5× ribosome concentration), and IF3 (1.5× ribosome concentration) in polymix buffer with varying addition of Mg(OAc)_2_ at 37°C for 15 min. Ternary complex mixture, containing ternary complexes EF-Tu·GTP·Glu-tRNA^Glu^, EF-Tu·GTP·Lys-tRNA^Lys^, or EF-Tu·GTP·Phe-tRNA^Phe^, was prepared by incubating the corresponding tRNA (tRNA^Glu^, tRNA^Lys^, or tRNA^Phe^; varying concentrations between 0.5 and 15 µM), EF-Tu (4 µM in excess over the highest tRNA concentration in a ternary complex titration experiment), amino acid (Glu, Lys, or Phe; 0.2 mM), aa-tRNA synthetase (GluRS, LysRS, or PheRS; 1.5 units/µL), and EF-Ts (1.5 µM) in polymix buffer with addition of varying concentrations of Mg(OAc)_2_ at 37°C for 15 min. Equal volumes of the ternary complex and ribosome mixtures were rapidly mixed in a temperature controlled quench-flow apparatus (RQF-3; KinTeck Corp.) (for cognate reactions) or manually (for near-cognate reactions). The reaction was stopped at different incubation times by rapidly quenching with formic acid (17% final concentration).

The extent of [^3^H]dipeptide formed at different time points was quantified by C18 reversed-phase HPLC equipped with a β-RAM model 3 radioactivity detector (IN/US Systems) (Johansson et al. 2012). The rate of cognate and near-cognate peptide bond formation (*k*_dip_^c/nc^) was estimated at different Mg^2+^ concentrations by fitting the data into a single exponential model:

$$\text{dip}(t)=[\text{Rib}]\cdot (1-e^{-k_{\mathrm{dip}}^{c/\mathrm{nc}}t})+\text{bg},$$

where dip(*t*) is the time evolution of dipeptide formed, and the plateau [Rib] is the active ribosome concentration in the reaction.

### Estimate of *k*_cat_/*K*_m_ for cognate and near-cognate dipeptide formation

For cognate reactions, we used *k*_cat_/*K*_m_-values for GTP hydrolysis from Zhang et al. (2015) as the *k*_cat_/*K*_m_-values for dipeptide formation, since there was no proofreading during tRNA selection (Thompson and Stone 1977; Ruusala et al. 1982; Gromadski and Rodnina 2004). As a control experiment, we only measured the *k*_cat_/*K*_m_-value for dipeptide formation for tRNA^Glu^ reading its cognate codon GAA at 2.3 mM free Mg^2+^ condition.

Ternary complexes, varied from 0.5 to 1.5 µM for cognate reaction and 0.5 to 15 µM for near-cognate reaction, are always in excess over ribosome complexes (0.2–0.5 µM); the rate of dipeptide formation was then limited by the concentration of ternary complexes [T_3_], and it follows Michaelis–Menten kinetics. At each Mg^2+^ concentration, the rate of cognate or near-cognate dipeptide formation *k*_dip_^c/nc^ was given by

$$k_{\mathrm{dip}}^{c/\mathrm{nc}}=\frac{{\left(k_{\mathrm{cat}}/K_{m}\right)}_{\mathrm{dip}}^{c/\mathrm{nc}}\cdot [T_{3}]}{1+([T_{3}]/K_{m})}.$$

The efficiency for cognate or near-cognate dipeptide formation ${\left(k_{\mathrm{cat}}/K_{m}\right)}_{\mathrm{dip}}^{c/\mathrm{nc}}$ and the *K*_m_-value were estimated by fitting *k*_dip_^c/nc^ to the experimental data.

### Estimate of total accuracy (*A*) and proofreading (*F*) factor

Total accuracy (*A*) is defined as the ratio between the kinetic efficiency for cognate and noncognate reaction for dipeptide formation.

$$A=\frac{{\left(k_{\mathrm{cat}}/K_{m}\right)}_{\mathrm{dip}}^{c}}{{\left(k_{\mathrm{cat}}/K_{m}\right)}_{\mathrm{dip}}^{\mathrm{nc}}}=I\cdot F.$$

The data of initial selection *I* were taken from Zhang et al. (2015). The proofreading parameter *F* was then calculated as *F* = *A*/*I*.

### Calibration of in vitro accuracy to the accuracy in vivo

Since different ternary complexes as well as release factors had very similar binding efficiency to the ribosome (Freistroffer et al. 2000; Johansson et al. 2011; Zhang et al. 2015), we calibrated our in vitro measurement according to the ratio of the abundance of tRNA^Glu^ and the tRNA cognate to the mismatch codon (Dong et al. 1996) or abundance of the release factors (Bremer and Dennis 2008) for measurement on stop codon UAA.

$$\text{Calibrated in vitro accuracy}=\frac{{\left(k_{\mathrm{cat}}/K_{m}\right)}_{\mathrm{dip}}^{c}}{{\left(k_{\mathrm{cat}}/K_{m}\right)}_{\mathrm{dip}}^{\mathrm{nc}}}\cdot \frac{{\left[{\mathrm{tRNA}}^{c}\;\mathrm{or}\;\mathrm{release}\;\;\mathrm{factors}\;1\;\mathrm{and}\;2\right]}^{\text{in vivo}}}{{\left[{\mathrm{tRNA}}^{\mathrm{Glu}}\right]}^{\text{in vivo}}}.$$

Error frequency is calculated as the inverse of the calibrated in vitro accuracy, which is shown as (▪) in Figure 3.

## SUPPLEMENTAL MATERIAL

Supplemental material is available for this article.

## Supplementary Material

Supplemental Material
