# Supplementary material for: Proofreading neutralizes potential error hotspots in genetic code translation by transfer RNAs
Source: RNA. 2016 Jun;22(6):896–904. doi: 10.1261/rna.055632.115 (PMC4878615; doi:10.1261/rna.055632.115)
Supplement: Supplemental Material [file supp_22_6_896__index.html]

Proofreading neutralizes potential error hotspots in genetic code translation by transfer RNAs — Proofreading neutralizes potential error hotspots in genetic code translation by transfer RNAs — Supplemental Material 

# Proofreading neutralizes potential error hotspots in genetic code translation by transfer RNAs

## Supplemental Material

- SuppMaterial.docx - docx file
- Supp\_TableS1.doc - docx file
