# Supplementary material for: Proofreading neutralizes potential error hotspots in genetic code translation by transfer RNAs
Source: RNA. 2016 Jun;22(6):896–904. doi: 10.1261/rna.055632.115 (PMC4878615; doi:10.1261/rna.055632.115)
Supplement: Supplemental Material [file supp_055632.115_SuppMaterial.docx]

**Supplementary material for**

**Proofreading neutralizes potential error hotspots in genetic code translation by transfer RNAs**

Jingji Zhang^1^, Ka-Weng Ieong^1^, Harriet Mellenius, and Måns Ehrenberg^*^

Department of Cell and Molecular Biology, Uppsala University, Husargatan 3, Box 596, Uppsala 75124, Sweden

^1^ Co-first authors

^*^ Corresponding author

Email: ehrenberg@xray.bmc.uu.se

Tel: +46 18 471 4213

Fax: +46 18 471 4262

**Part I: Statistical analysis of the correlation of initial selection and proofreading**

The proofreading selection (*F*) was plotted against the initial selection (*I*) in Fig. 5A in the main text, both of them logarithm transformed with the base 10. It seems like there is a positive linear correlation between the transformed initial selection and proofreading for high values of initial selection, but that the proofreading selection is constant when the initial selection decreases below a value of about 3,000.

To test this hypothesis, we fitted the relation of the two logarithm transformed selections to four different models. The first model is that the proofreading data can be described by a constant value, the average of log_10_(*F*) = 2.043 (Fig. S1 A). In model number 2 the variation in the proofreading data is explained by a straight line, fitted to

$$\text{log}_{10}\left(F\right)\text{ }=\text{ }0.59\times\text{log}_{10}\left(I\right)+0.021$$

(Fig S1 B). In the third model, the variation in the data is explained by a constant *and* a straight line, fitted to

$$\text{log}_{10}\left(F\right)\text{ }=\text{ max}\left(1.7,\text{ 1}.6\times\text{log}_{10}\left(I\right)-4.1\right)$$

(Fig S1 C, Fig 5 A). In the fourth model the variation in the data is explained by two straight lines, fitted to

$$\text{log}_{10}\left(F\right)\text{ }=\text{ max}\left(-0.031\times\text{log}_{10}\left(I\right)+1.8,\text{ 1}.6\times\text{log}_{10}\left(I\right)-3.9\right)$$

(Fig S3 D).

The intercept of the constant and the straight line in model 3 occurs at log_10_(*I*) = 3.54 and of the two straight lines in model 4 at log_10_(*I*) = 3.52. All models were fitted using the least squares approach.


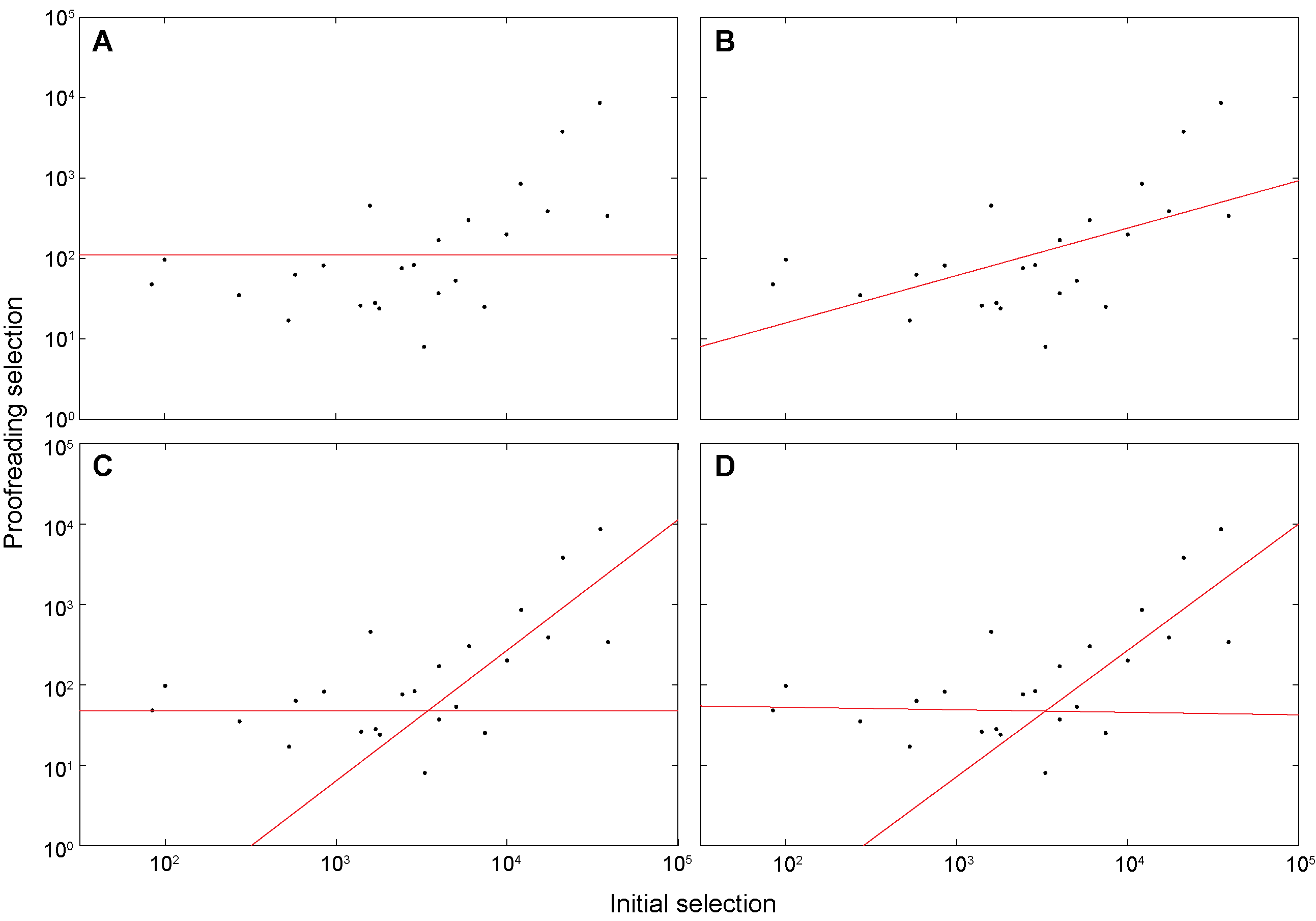


**Figure S1. Scatter plot of the logarithm transformed proofreading selection against the logarithm transformed initial selection.** The data are fitted to four different models, where y = log_10_(*F*) and x = log_10_(*I*): (A) y = 2.0. (B) y = 0.59*x + 0.021. (C) y = max(1.7, 1.6*x - 4.1). (D) y = max(-0.031*x + 1.8, 1.6*x - 3.9).

To test the models against each other, an *F*-test statistic was formed and compared to the *F*-distribution. Firstly, we tested the hypothesis that the variation in the data is just as well described by a constant (the null hypothesis) as by a straight line (Eq. 1). The *F*-test statistic was calculated as

$F=\frac{\text{SS}_{\mathrm{mod}2}}{\text{SS}_{res(\mathrm{mod}2)}}/\frac{\text{df}{}_{\mathrm{mod}2}}{\text{df}{}_{res(\mathrm{mod}2)}}$,

where SS_mod_, the sum of squares of the model, is the sum of squares of the deviations of the modeled data about the mean of the response variable, the logarithm transformed proofreading selection, SS_res_ is the sum of squares of residuals and df is the degrees of freedom. The degrees of freedom of the residuals is the total degrees of freedom minus the degrees of freedom of the model, here 24-2=22. The *p*-value for this *F*-test with the degrees of freedom of 2 and 22, respectively, was 0.011. Thus, the null hypothesis that the variation in the log-transformed proofreading data does not depend linearly on the log-transformed initial selection data can be rejected at a significance level of 0.05.

Next, we tested if the variation in the data is just as well described by a straight line as by a constant and a straight line, the data fitted to give the function in Eq. 2. The *F*-test statistic was calculated as

$$F=\frac{\text{SS}_{\mathrm{mod}3}-\text{SS}_{\mathrm{mod}2}}{\text{SS}_{res(\mathrm{mod}3)}}/\frac{\text{df}{}_{\mathrm{mod}3}-\text{df}{}_{\mathrm{mod}2}}{\text{df}{}_{res(\mathrm{mod}3)}}$$

The *p*-value for this *F*-test with the degrees of freedom of 1 (the difference in degrees of freedom of the two models) and 24-3=21, respectively, was 0.0019. The hypothesis that the variation in the data is better or just as well described by a straight line as by a constant and a straight line can thus be rejected.

Lastly, we tested if the variation in the log_10_(*F*) data is just as well described by a constant and a straight line as by two straight lines, the data fitted to give the function in Eq. 3. The *F*-test statistic was calculated

$$F=\frac{\text{SS}_{\mathrm{mod}4}-\text{SS}_{\mathrm{mod}3}}{\text{SS}_{res(\mathrm{mod}4)}}/\frac{\text{df}{}_{\mathrm{mod}4}-\text{df}{}_{\mathrm{mod}3}}{\text{df}{}_{res(\mathrm{mod}4)}}$$

The *p*-value for this *F*-test with the degrees of freedom of 1 and 20, respectively, was 1, since the model sum of squares is actually higher for model 3 than for model 4. It might seem surprising that the explained sum of squares (the sum of squares of the model) is lower for model 4 as it has a higher number of degrees of freedom. However, this is possible since the fitting minimizes the residuals, which in this case brings the model slightly closer to the mean, which is what the sum of squares of the model measures. The fact that model 4 is better in terms of explained variance (*R^2^*, Table S2) and model 3 in terms of the model sum of squares implies the two models can be viewed as almost equally good, but with a lower number of degrees of freedom in model 3. This is underscored by the confidence intervals of the fitted parameters of model 3 and model 4 that overlap very much (Table S3). The hypothesis that the variation in the data is better or just as well described by two straight lines as by a constant and a straight line cannot be rejected at any significance level.

The models and their related adjusted *R^2^* and σ-values are also presented in Table S2, along with the *p*-values for each model tested against the previous one. The adjusted *R^2^*, calculated as

$aR^{2}=1-\frac{\text{SS}_{res}}{\text{SS}_{tot}}/\frac{\text{df}_{res}}{\text{df}{}_{tot}}$,

is the fraction of the variance in the data that is explained by the model adjusted for degrees of freedom.

The adjusted *R^2^* and the *F*-tests both suggest that the best model to represent the relation between the logarithm transformed initial selection and proofreading selection is a constant and a straight line, in accordance with the biological interpretation.

**Table S2. The four models, their adjusted *R^2^* and the p-values of each model tested against the previous.**

| **Model equation** | ***R^2^*** | **Adjusted *R^2^*** | **p-value** |
| --- | --- | --- | --- |
| y = 2.0 | 0 |  |  |
| y = 0.59*x + 0.021 | 0.337 | 0.307 | 0.0108 |
| y = max(1.7, 1.6*x - 4.1) | 0.586 | 0.547 | 0.00187 |
| y = max(-0.031*x + 1.8, 1.6*x - 3.9) | 0.587 | 0.525 | 1 |

**Table S3. Fitted parameters with σ-values for models 3 and 4.**

| **Model** | **Parameter value** | **σ** |
| --- | --- | --- |
| y = max(a, b*x + c) | a = 1.7 | 8.4% |
|  | b = 1.6 | 28% |
|  | c = -4.1 | 44% |
| y = max(d*x + e, f*x + g) | d = -0.031 | 903% |
|  | e = 1.8 | 47% |
|  | f = 1.6 | 29% |
|  | g = -3.9 | 48% |

**Part II: Tunable accuracy of tRNA selection**


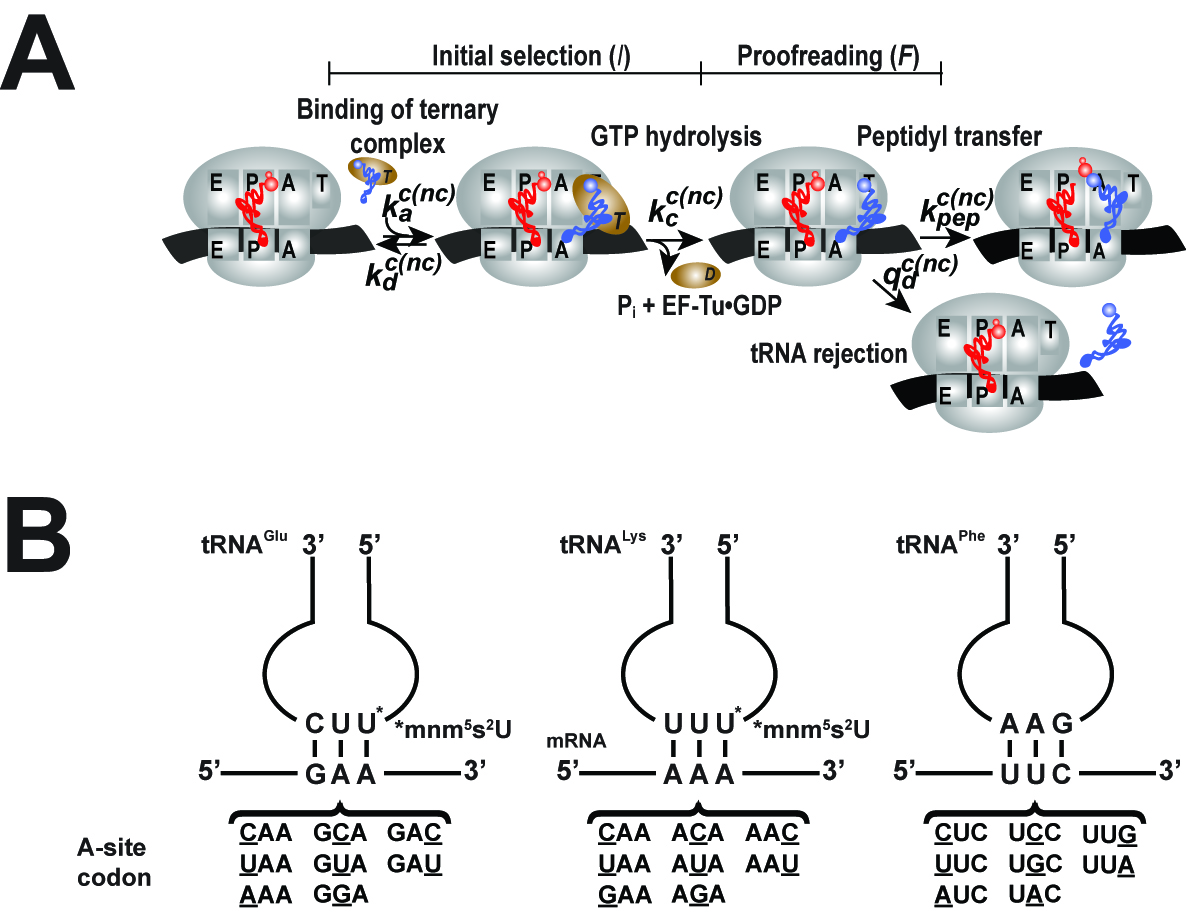


**Figure S2. Kinetic scheme of tRNA selection on the mRNA programmed ribosome.** A ternary complex bind to the ribosome with the association rate constant *k*_a_ and dissociation constant *k*_d_. After that, GTP is hydrolyzed on EF-Tu with the rate constant *k*_c_. Then the tRNA either proceeds accommodation and peptidyl transfer with the rate constant *k*_pep_ or is rejected by the ribosome with the discard rate constant *q*_d_. The notations *c* and *nc* represent cognate and non-cognate reactions, respectively.

From scheme in Fig. S2, initial selection can be expressed as:

$$I=\frac{(k_{cat}/K_{m})_{GTP}^{c}}{(k_{cat}/K_{m})_{GTP}^{nc}}=\frac{k_{a}^{c}}{k_{a}^{nc}}\times\frac{1+k_{d}^{nc}/k_{c}^{nc}}{1+k_{d}^{c}/k_{c}^{c}}=\frac{1+d_{I}\cdot a_{I}}{1+a_{I}}$$

where cognate and non-cognate ternary complexes have the same association rate constants for binding to the ribosome (Gromadski and Rodnina 2004; Gromadski et al. 2006), $k_{a}^{nc}=k_{a}^{c}$ . Here we define $d_{I}=\frac{k_{d}^{nc}/k_{c}^{nc}}{k_{d}^{c}/k_{c}^{c}}$and $a_{I}=k_{d}^{c}/k_{c}^{c}$, where *d_I_* is the intrinsic discrimination parameter for initial selection, and *a_I_* is the discarding parameter for initial selection. The cell can tune initial selection by varying *a_I_*. We previously estimated $a_{I}\approx1$ under vivo-like conditions (2.3 mM free Mg^2+^ in polymix) (Johansson et al. 2012; Zhang et al. 2015) . From Eq. 8, we get:

$$I=\frac{1+d_{I}\cdot a_{I}}{1+a_{I}}\Rightarrow d_{I}\approx2I$$

Total accuracy can be expressed as:

$$A=\frac{(k_{cat}/K_{m})_{dip}^{c}}{(k_{cat}/K_{m})_{dip}^{nc}}=\frac{k_{a}^{c}}{k_{a}^{nc}}\times\frac{1+k_{d}^{nc}/k_{c}^{nc}}{1+k_{d}^{c}/k_{c}^{c}}\times\frac{1+q_{d}^{nc}/k_{pep}^{nc}}{1+q_{d}^{c}/k_{pep}^{c}}$$

Proofreading can be expressed as:

$$F=A/I=\frac{1+q_{d}^{nc}/k_{pep}^{nc}}{1+q_{d}^{c}/k_{pep}^{c}}=\frac{1+d_{F}\cdot a_{F}}{1+a_{F}}$$

where $d_{F}=\frac{q_{d}^{nc}/k_{pep}^{nc}}{q_{d}^{c}/k_{pep}^{c}}$ and $a_{F}=q_{d}^{c}/k_{pep}^{c}$. Here, *d_F_* is the intrinsic discrimination parameter for proofreading, and *a_F_* is the discarding parameter for proofreading.

Assuming the intrinsic discrimination parameter for proofreading selection to be the same as for initial selection,$d_{F}=d_{I}$, it follows from Eq. 11 that:

$$F=\frac{1+d_{F}\cdot a_{F}}{1+a_{F}}=\frac{1+d_{I}\cdot a_{F}}{1+a_{F}}\approx\frac{d_{I}\cdot a_{F}}{1+a_{F}}$$

From Eq. 9 $d_{I}\approx2I$, we get :

$$F\approx\frac{a_{F}2I}{1+a_{F}}\approx a_{F}2I$$

From Eq. 13 follows that when $a_{F}$ << 1 and constant, log(*F*) is decreases linearly with decreasing log(*I*). When initial selection is below a certain limit then, by hypothesis, there is strong counter selection against further decrease in the total accuracy, *A*, so that the discard parameter *a_F_* increases with the consequence that *F* decreases insignificantly with decreasing *I* in the low accuracy region. This means, in other words, that although the intrinsic proofreading accuracy (*d_F_*=*d_I_*) decreases, its expressed fraction *a_F_*/(1+*a_F_*) increases so that *F* remains virtually constant. Intriguingly, the data in Fig. 5A suggest that log(*F*) decreases more rapidly with log(*I*) in the high accuracy range than suggested by Eq. 13, since the line has a slope of 1.6 and not one. This could mean a more complex proofreading mechanism than the one depicted in Fig. S2 and Fig.1 (in the main text), e.g. involving more than one discard step (Ehrenberg and Blomberg 1980), but the explanation for the near-constant *F*-value would still be valid.

**Supplementary references:**

Ehrenberg M, Blomberg C. 1980. Thermodynamic constraints on kinetic proofreading in biosynthetic pathways. *Biophys J* **31**(3): 333-358.

Gromadski KB, Daviter T, Rodnina MV. 2006. A uniform response to mismatches in codon-anticodon complexes ensures ribosomal fidelity. *Mol Cell* **21**(3): 369-377.

Gromadski KB, Rodnina MV. 2004. Kinetic determinants of high-fidelity tRNA discrimination on the ribosome. *Mol Cell* **13**(2): 191-200.

Johansson M, Zhang JJ, Ehrenberg M. 2012. Genetic code translation displays a linear trade-off between efficiency and accuracy of tRNA selection. *Proceedings of the National Academy of Sciences of the United States of America* **109**(1): 131-136.

Zhang J, Ieong KW, Johansson M, Ehrenberg M. 2015. Accuracy of initial codon selection by aminoacyl-tRNAs on the mRNA-programmed bacterial ribosome. *Proceedings of the National Academy of Sciences of the United States of America* **112**(31): 9602-9607.
