# Supplementary material for: Proofreading neutralizes potential error hotspots in genetic code translation by transfer RNAs
Source: RNA. 2016 Jun;22(6):896–904. doi: 10.1261/rna.055632.115 (PMC4878615; doi:10.1261/rna.055632.115)
Supplement: Supplemental Material [file supp_055632.115_Supp_TableS1.doc]

| **Table S1.** *k_cat_/K_m_*-values for peptide bond formation from different tRNAs reading their cognate and near-cognate codons. Measurements were performed at 37 °C in polymix buffer with different free Mg^2+^ concentration. Data are presented as the weighted average from two or more experiments ± the propagated standard deviation. | | | | | | | | | | | | | | | | |  | |
| --- | --- | --- | --- | --- | --- | --- | --- | --- | --- | --- | --- | --- | --- | --- | --- | --- | --- | --- |
|  |  | Free Mg^2+^ concentration (mM) | | | | | | | | | | | | | | |  | |
|  |  | 1.3 | | | 2.3 | | | 3.4 | | | 4.6 | | | 7.5 | | |  | |
| tRNA | Codon | kcat/Km  (µM^-1^s^-1^) | kcat  (s^-1^) | Km  (µM) | kcat/Km  (µM^-1^s^-1^) | kcat  (s^-1^) | Km  (µM) | kcat/Km  (µM^-1^s^-1^) | kcat  (s^-1^) | Km  (µM) | kcat/Km  (µM^-1^s^-1^) | kcat  (s^-1^) | Km  (µM) | kcat/Km  (µM^-1^s^-1^) | kcat  (s^-1^) | Km  (µM) |  | |
| $tRNA_{UUU}^{Lys}$ | AAA^*^ | 60±4 | - | - | 117±9 | - | - | 147±7 | - | - | 167±14 | - | - | 180±18 | - | - |  | |
|  | CAA | - | - | - | 6.35×10^-5^±9.00×10^-6^ | 1.72×10^-4^±7.80×10^-5^ | 2.71±1.17 | - | - | - | - | - | - | - | - | - |  | |
|  | UAA | - | - | - | 2.42×10^-3^±1.92×10^-4^ | 2.18×10^-2^±1.34×10^-2^ | 9.01±5.49 | 1.37×10^-2^±1.64×10^-3^ | 1.40×10^-2^±4.71×10^-3^ | 1.02±3.22×10^-1^ | - | - | - | - | - | - |  | |
|  | GAA | 3.90×10^-4^±3.00×10^-5^ | 5.57×10^-3^±2.77×10^-3^ | 14.3±7.0 | 2.73×10^-3^±2.30×10^-4^ | 9.82×10^-3^±2.53×10^-3^ | 3.60±0.88 | 9.86×10^-3^±1.61×10^-3^ | 1.68×10^-2^±7.68×10^-3^ | 1.71±0.73 | 3.67×10^-2^±6.79×10^-3^ | 2.46×10^-2^±8.93×10^-3^ | 6.71×10^-1^±2.09×10^-1^ | 2.50×10^-1^±7.18×10^-2^ | 3.44×10^-2^±1.56×10^-2^ | 1.38×10^-1^±4.84×10^-2^ |  | |
|  | ACA | - | - | - | 5.67×10^-5^±3.17×10^-6^ | - | - | - | - | - | - | - | - | - |  |  |  | |
|  | AUA | - | - | - | 1.80×10^-4^±1.63×10^-5^ | 6.97×10^-4^±2.29×10^-4^ | 3.87±1.22 | 5.35×10^-4^±3.23×10^-5^ | 4.01×10^-3^±2.00×10^-3^ | 7.48±3.72 | - | - | - | - | - | - |  | |
|  | AGA | - | - | - | 4.44×10^-3^±3.19×10^-4^ | - | - | 1.67×10^-2^±1.06×10^-3^ | 8.50×10^-2^±3.92×10^-2^ | 5.10±2.33 | 5.62×10^-2^±7.94×10^-3^ | 8.59×10^-2^±4.19×10^-2^ | 1.53±7.13×10^-1^ | - | - | - |  | |
|  | AAC | - | - | - | 3.36×10^-3^±1.44×10^-4^ | 2.32×10^-2^±6.64×10^-3^ | 6.91±1.96 | 1.12×10^-2^±3.56×10^-4^ | - | - | 3.94×10^-2^±3.01×10^-3^ | 8.75×10^-2^±2.83×10^-2^ | 2.22±6.97×10^-1^ | - | - | - |  | |
|  | AAU | - | - | - | 1.30×10^-2^±4.11×10^-4^ | - | - | - | - | - | - | - | - | - | - | - |  | |
| $tRNA_{UUC}^{Glu}$ | GAA^*^ | 51±4 | - | - | 91±8 | - | - | 117±7 | - | - | - | - | - | 144±9 | - | - |  | |
|  | CAA | - | - | - | 3.01×10^-7^±1.08×10^-8^ | - | - | 1.06×10^-6^±1.94×10^-7^ | - | - | - | - | - | 6.49×10^-6^±3.14×10^-6^ | - | - |  | |
|  | UAA | - | - | - | 1.13×10^-6^±5.17×10^-8^ | - | - | - | - | - | - | - | - | - | - | - |  | |
|  | AAA | - | - | - | 1.34×10^-5^±4.5×10^-7^ | 2.55×10^-5^±8.11×10^-6^ | 1.90±0.60 | - | - | - | - | - | - | - | - | - |  | |
|  | GCA | - | - | - | 8.84×10^-6^±2.12×10^-7^ | - | - | 2.48×10^-5^±8.17×10^-6^ | 1.12×10^-4^±1.11×10^-4^ | 4.50±4.20 | - | - | - | - | - | - |  | |
|  | GUA | - | - | - | 1.25×10^-4^±8.92×10^-6^ | - | - | - | - | - | - | - | - | - | - | - |  | |
|  | GGA | 2.34×10^-3^±1.60×10^-4^ | 1.68×10^-2^±3.69×10^-3^ | 7.20±1.50 | 2.23×10^-2^±1.07×10^-3^ | - | - | 1.37×10^-1^±1.75×10^-2^ | 3.85×10^-1^±1.85×10^-1^ | 2.80±1.30 | - | - | - | 4.27±1.27 | 2.09±1.13 | 0.49±0.22 |  | |
|  | GAC | 2.13×10^-3^±3.50×10^-4^ | 1.24×10^-2^±1.21×10^-2^ | 5.80±5.60 | 9.50×10^-3^±5.13×10^-4^ | - | - | 3.54×10^-2^±2.27×10^-3^ | - | - | - | - | - | 4.73×10^-1^±6.39×10^-2^ | - | - |  | |
|  | GAU | 1.17×10^-3^±1.98×10^-4^ | - | - | 9.57×10^-3^±3.67×10^-4^ | - | - | 6.59×10^-2^±4.88×10^-3^ | - | - | - | - | - | 4.21±1.21 | 3.41±2.06 | 0.81±0.43 |  | |
| $tRNA_{GAA}^{Phe}$ | UUC^*^ |  |  |  | 76±5 |  |  |  |  |  |  |  |  |  |  |  |  |  |
|  | CUC | - | - | - | 1.09×10^-3^±5.22×10^-5^ | 3.32×10^-3^±8.05×10^-4^ | 3.05±0.72 | - | - | - | - | - | - | - | - | - |  | - |
|  | GUC | - | - | - | 4.08×10^-4^±3.37×10^-5^ | 1.06×10^-4^±1.58×10^-5^ | 0.26±0.03 | - | - | - | - | - | - | - | - | - |  | - |
|  | AUC | - | - | - | 2.81×10^-4^±2.18×10^-5^ | 1.00×10^-4^±1.39×10^-5^ | 0.36±0.04 | - | - | - | - | - | - | - | - | - |  | - |
|  | UCC | - | - | - | 3.18×10^-4^±2.90×10^-5^ | 2.49×10^-4^±5.42×10^-5^ | 0.78±0.16 | - | - | - | - | - | - | - | - | - |  | - |
|  | UGC | - | - | - | 5.67×10^-6^±3.33×10^-7^ | - | - | - | - | - | - | - | - | - | - | - |  | - |
|  | UAC | - | - | - | 4.12×10^-4^±5.78×10^-5^ | 7.07×10^-5^±1.58×10^-5^ | 0.17±0.03 | - | - | - | - | - | - | - | - | - |  | - |
|  | UUG | - | - | - | 5.13×10^-4^±3.35×10^-5^ | 9.72×10^-4^±2.01×10^-4^ | 1.89±0.37 | - | - | - | - | - | - | - | - | - |  | - |
|  | UUA | - | - | - | 2.07×10^-3^±5.30×10^-5^ | - | - | - | - | - | - | - | - | - | - | - |  | - |
| ^*^ Data shown here are *k_cat_*/*K_m_*-values for GTP hydrolysis on EF-Tu under the same experimental condition from (Zhang et al., 2015). Since there is no proofreading in cognate reactions, *k_cat_*/*K_m_*-values for peptide bond formation are equal to *k_cat_*/*K_m_*-values for GTP hydrolysis. | | | | | | | | | | | | | | | | |  | |
